# Prevalence, serotypes and virulence genes of *Streptococcus agalactiae* isolated from pregnant women with 35–37 weeks of gestation

**DOI:** 10.1186/s12879-020-05603-5

**Published:** 2021-01-14

**Authors:** Fernando J. Bobadilla, Marina G. Novosak, Iliana J. Cortese, Osvaldo D. Delgado, Margarita E. Laczeski

**Affiliations:** 1 Facultad de Ciencias Exactas, Químicas y Naturales (FCEQyN), Departamento de Microbiología, Universidad Nacional de Misiones (UNaM), Consejo Nacional de Investigaciones Científicas y Técnicas (CONICET), Posadas, Argentina; 2 Facultad de Ciencias Exactas, Químicas y Naturales (FCEQyN), Instituto de Biotecnología de Misiones (InBioMis), Universidad Nacional de Misiones (UNaM), Consejo Nacional de Investigaciones Científicas y Técnicas (CONICET), Posadas, Argentina; 3 Facultad de Ciencias Exactas y Naturales (FACEN), Universidad Nacional de Catamarca (UNCa), Centro de Investigación y Transferencia Catamarca (CITCA-CONICET), San Fernando del Valle de Catamarca, Argentina

**Keywords:** GBS, Colonization, Serotypes, Virulence genes

## Abstract

**Background:**

In pregnant women *Streptococcus agalactiae* (GBS) can be transmitted to newborn causing severe infections. It is classified into 10 serotypes (Ia, Ib, II-IX). The severity of neonatal disease is determined by the capsular serotype and virulence factors such as the polysaccharide capsule, encoded by the *cps* gene, protein C, which includes the Cα surface proteins (*bca* gene), Rib (*rib* gene) and Cβ (*bac* gene); the proteins Lmb (*lmb* gene), FbsB (*fbsB* gene), FbsA (*fbsA* gene), the *cyl* operon encoding a β-hemolysin *(hylB* gene), the CAMP factor (*cfb* gene) and the C5a peptidase (*scpB* gene). The aim of this work was to determine the degree of GBS colonization in pregnant women, the serotypes distribution and to investigate virulence-associated genes.

**Methods:**

We worked with 3480 samples of vagino-rectal swabs of women with 35–37 weeks of gestation. The identification of the strains was carried out using conventional biochemical tests and group confirmatory serology using a commercial latex particle agglutination kit. Two hundred GBS strains were selected. Their serotype was determined by agglutination tests. The monoplex PCR technique was used to investigate nine virulence-associated genes (*cps*, *bca*, *rib*, *bac*, *lmb*, *fbsB*, *fbsA*, *hy*l*B* and *scpB*).

**Results:**

The maternal colonization was 9.09%. The serotypes found were: Ia (33.50%), III (19.00%), Ib (15.50%), II (14.00%), V (7.00%) and IX (5.50%). 5.50% of strains were found to be non-serotypeable (NT). The nine virulence genes investigated were detected simultaneously in 36.50% of the strains. The genes that were most frequently detected were *scpB* (100.00%), *fbsA* (100.00%), *fbsB* (100.00%), *cylB* (95.00%), *lmb* (94.00%) and *bca* (87.50%). We found associations between serotype and genes *bac* (*p* = 0.003), *cylB* (*p* = 0.02), *rib* (*p* = 0.01) and *lmb* (*p* < 0.001).

**Conclusions:**

The frequency of vaginal-rectal colonization, serotypes distribution and associated virulence genes, varies widely among geographical areas. Therefore, epidemiological surveillance is necessary to provide data to guide decision-making and planning of prevention and control strategies.

## Background

*S. agalactiae*, Group B *Streptococcus* (GBS), is one of the main causes of morbidity and mortality in neonates and severe infections in pregnant women and in nonpregnant adults especially among patients with underlying medical conditions, such as Diabetes Mellitus or immunosuppression [1].

*S. agalactiae* forms part of the human intestinal microbiota, from which colonizes the genital tract, which allows the transmission to the newborn. To prevent neonatal infection, the Center for Disease Prevention and Control (CDC) recommends testing for GBS in all pregnant women between 35 and 37 weeks of gestation by vaginal (anterior third) and anorectal swabbing [2].

In 2007 and 2008, in Misiones Province and Argentina, Laws XVII - N° 59 and 26,369 were passed, that adhere to the mandatory search for GBS in pregnant women, and, detected maternal colonization, to the implementation of intrapartum antibiotic prophylaxis (IPP) [3, 4].

*S. agalactiae* produces severe infections such as septicemia, pneumonia and meningitis [5]. In addition, it is an important cause of infection in pregnant and puerperal women producing chorioamnionitis, postpartum endometritis, post-cesarean surgical wound infection and urinary tract infection [6, 7].

Lancefield defined two types of carbohydrate antigens in the GBS wall. The group B antigen, common to all strains, and the specific capsular polysaccharide antigen that allows its classification into 10 serotypes: Ia, Ib, II, III, IV, V, VI, VII, VIII and IX, with different geographic distribution [8].

The severity of neonatal disease is largely determined by the capsular serotype and virulence factors; they are necessary for host-bacterial cell interaction [9].

The main surface structures of GBS involved in virulence are:

Protein C, which includes the surface proteins Cα (*bca* gene), Rib (*rib* gene) and Cβ (*bac* gene); mediates adhesion to host cells [10, 11]. The *lmb* gene codes for the Lmb protein that participates in the adhesion to laminin of the extracellular matrix of the host cell, especially the placental membrane, which also facilitates the invasion of the endothelial cells of the central nervous system [12]. The protein of adherence to fibrinogen FbsB, which adheres to lung epithelial cells and protects the bacteria from opsonization in the human bloodstream, is encoded by the *fbs*B gene [13].

The *cyl* operon encodes a β-hemolysin that is a toxin associated with tissue injury and systemic spread contributing to meningitis [14]. The *hyl*B gene encodes Hyaluronidate lyase, which is an essential component to allow the bacteria to spread from the initial site of infection [15]. The CAMP factor, which is present in all GBS isolates, is encoded by the *cfb* gene. It has the property of lysing the membranes of erythrocytes that have been pre-treated with the β-hemolysin of *Staphylococcus aureus*, a sphingomyelinase [16].

The polysaccharide capsule, encoded by the *cps* gene, which prevents the elimination of pathogens by the immune system mainly by two mechanisms; prevents the deposit of complement and phagocytosis [17]. Protein FbsA, encoded by the *fbs*A gene, protects the bacterium from opsono-phagocytosis and promotes its adhesion to the epithelial cells and especially the cerebral endothelium, helping the pathogen to cross the blood-brain barrier leading to meningitis [18].

C5a peptidase, encoded by the *scp*B gene, is a serine protease that inactivates human C5a (a chemotactic protein). Thus, it inhibits the recruitment of neutrophils and helps reduce the inflammatory response of the host [19].

The frequency of vaginal-rectal colonization varies according to geographic area, ethnic and social conditions in populations [20]. The province of Misiones has the particularity that ethnic origins are varied, mainly from European immigrants and from neighboring countries. Unlike the rest of the country, the province of Misiones shares more than 80% of its borders with Paraguay and Brazil. In addition, 73.76% of the population lives in urban areas, 26.24% lives in rural areas, 95.89% is literate population and 27.80% is poor [21, 22].

In 2013, Oviedo et al characterized the GBS colonization rate, serotypes distribution, resistance phenotypes and five genes associated with virulence in pregnant women screened between 2004 and 2010 in the province of Misiones. They established that maternal colonization was 9.38%, serotype Ia was the most frequent, whereas serotypes IV, VI, VII and VIII were not detected. Resistance to erythromycin was 11.6% and the *lmb*, *bca* and h*ylB* genes were detected in more than 79% of the strains [23].

The importance of investigating and maintaining the surveillance of serotypes and virulence factors in circulating strains in a region, lies in the fact that they allow the development of more effective multivalent maternal vaccines to improve the prevention of the disease caused by GBS [24, 25]. As well as, to identify potential outbreaks and hypervirulent clones due to the potential for GBS to disseminate in hospital settings [26].

The surveillance could also inform whether there are temporal changes in serotype distribution. There is a paucity of longitudinal data on the serotype distribution of group B *Streptococcus* (GBS) from low-middle income countries, which could inform selection of vaccine epitopes [27].

In this context, to contribute to the low amount of national and regional data, the objective of this research was to determine the degree of GBS colonization in pregnant women, the distribution of serotypes, and to investigate nine virulence-associated genes.

## Methods

### Ethics approval and informed consent

This work was approved by the Scientific Committee of the Central Hospital ‘Dr. Ramón Madariaga’ of the city of Posadas, Misiones, under the title: ‘Study of Bacterial and Perinatological Infections in the Province of Misiones’.

Written informed consent was obtained from each patient and confidential medical data according to study protocol: C10 ‘Prevalence of vaginal and rectal colonization of Streptococcus Beta-hemolytic group B (GBS or *S. agalactiae*) in pregnant women of 35-37 weeks of gestation’.

### Colonization in full-term pregnant women

Within January 2004 to December 2014, 3480 samples of vagino-rectal swabs were taken from women 35–37 weeks of gestational age with an average of 316 samples per year, concurrent to the Central Hospital “Dr. Ramón Madariaga” and to peripheral Primary Health Care Centers in Posadas City and from different areas of the province of Misiones (Garupá, Apóstoles, Aristóbulo del Valle, Oberá, Eldorado and Iguazú).

The samples were taken from the anterior third of the vagina with a swab and simultaneously with another swab from the ano-rectal area. The patients did not receive antimicrobials days before and during the sampling.

The vagino-rectal swab was seeded in 1–2 mL of Todd-Hewitt broth supplemented with colistin (10 μg mL^− 1^) and nalidixic acid (15 μg mL^− 1^). The sown stock was grown 18–24 h in an incubator at 35 °C.

After incubation, it was transferred to a 5% sheep blood agar plate with seeding technique for isolation. The plates were incubated in micro-aerobic atmosphere for 24 h. Suspicious β or γ hemolytic colonies were taken from the plates.

**Fig. 1:**
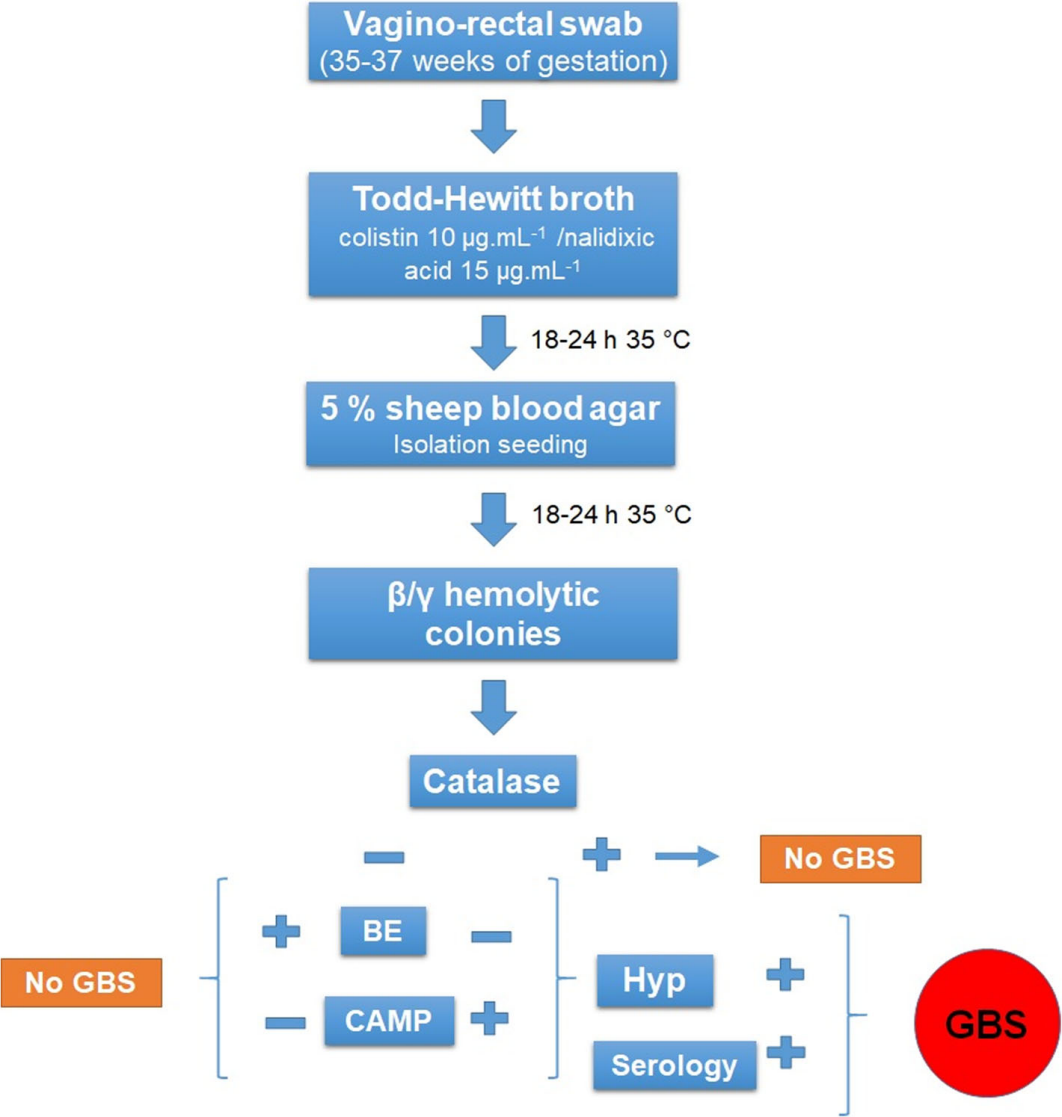
Isolation and identification algorithm of *S. agalactiae* (GBS). BE: Bile Esculin; CAMP: CAMP factor test; Hyp: Hydrolysis of hippurate; Serology: Agglutination test for group confirmation with sera Phadebact Strep B Test-ETC international-Bactus AB, Sweden

The bacterial identification was performed by conventional biochemical tests. The group confirmatory serology was performed using a commercial latex particle agglutination kit (Phadebact Strep B Test-ETC International-Bactus AB, Sweden), according to the recommendations of the manufacturer (Fig. 1).

A uniform stratified sampling was performed: each year was defined as a single stratum; the sample size is the similar in all strata (18 per year except for differences due to necessary rounding). Therefore, two hundred GBS strains were selected to perform serotype determination and to investigate nine virulence-associated genes.

### Serotype determination

The agglutination test of the Statens Serum Institut (Strep-B Latex, Copenhagen, Denmark) was used, which contains 10 serotypes (Ia, Ib, II to VIII and IX), following the instructions of the manufacturer for the performance of the test. A single colony was taken, blood agar was re-seeded and, from this plate, serotype and gene detection tests were carried out.

### Molecular detection of virulence-associated genes

The virulence genes: *bac*, *bca* and *rib* (components of protein C), *lmb* (Protein Lmb), *hylB* (hyaluronidate lyase), *scp*B (C5a peptidase), *cyl*B (Hemolysin), *fbs*A (Protein FbsA) and *fbs*B (Protein FbsB) were investigated using monoplex PCR technique. The genes studied were chosen because they encode virulence factors involved during the development of neonatal infection.

The primers used to search for virulence genes were designed with the program *Primer3* version 0.4.0 [28] (Table 1).

**Table 1:** Primers used to amplify virulence-associated genes in *S. agalactiae* strains

Genes	Forward 5′-3′	Reverse 5′-3′	Amplicon (bp)	TM °C
*bac*	TGTAAAGGACGATAGTGTGAAGAC	CATTTGTGATTCCCTTTTGC	530	50
*bca*	CAGGAAGGGGAAACAACAGTAC	GTATCCTTTGATCCATCTGGATACG	535	50
*rib*	CAGGAAGTGCTGTTACGTTAAAC	CGTCCCATTTAGGGTCTTCC	369	50
*hylB*	TTATCATCCAGCGCCTCCTAG	GTGGTGATAACTGACTTCTTGGGA	245	50
*lmb*	GACGCAACACACGGCAT	TGATAGAGCACTTCCAAATTTG	300	50
*scpB*	AGCCATATGCTGCGATCTCT	GGGTTGAACCAAGTGTGCTT	198	58
*cylB*	GGGCTGCAGGTATTATCGAA	ATTTCCACCAAAAGCAAACG	176	58
*fbsA*	TGTAGCTAATGGACCGATGTT	TTTTCATTGCGTCTCAAACC	156	58
*fbsB*	ACAACTGCGGAAATGACCTC	ACGAGCGACGTTGAATTCTT	186	58

*bp* base pairs

The sequences were aligned using BlastN algoritm, available at NCBIk and primers were synthesized by *Operon Molecules for Life* (USA): *bac, bca, rib, hylB, lmb,* and by *Macrogen* (South Korea) *scpB, cylB, fbsA* and *fbsB*.

An internal positive control, obtained by sequencing, was used for all virulence-associated genes investigated.

The chromosomal DNA extraction was carried out according to the protocol of Sambrook and Russell (2001) [29] modified by Cariaga Martinez y Zapata (2007) [30]. The PCR was performed with 20 ng of DNA in a final volume of 20 μL containing 1X of TaqDNA polymerase buffer (10X: 500 mM KCl, 100 mM Tris-HCl, pH 9.0 a 25 °C, 1% Triton®X-100), 200 μM of each of the dNTPs, 10 pmol of each primer and 0.5 U of the enzyme TaqDNA polymerase (Inbio Highway®, Argentina).

The cycling program was as follow: pre-denaturation at 94 °C for 2 min, followed by 30 cycles (30 s at 94 °C, 60 s at 50 °C, 60 s at 72 °C) and a final elongation at 72 °C for 2 min, in a Multigene TM II thermocycler (Labnet International Inc., USA).

The obtained amplicons were visualized on 2% agarose gels (m/v). The electrophoretic run was performed in an electrophoretic cell (Electrophoresis Subsistem 70 Labnet International) at 100 V for 60 min and subsequent observation of the bands in UV transilluminator (Model MUV 21–312-220). It was photographed with Canon Power Shot G10 digital camera.

### DNA sequencing

The sequencing of the PCR products was carried out for the *rib* gene by the Sequencing Service of the University of Buenos Aires (UBA) and for the *bac*, *bca*, *lmb*, *hyl*B, *scp*B, *cyl*B, *fbs*A and *fbs*B genes; through the Automatic Sequencing Service *Macrogen* inc., South Korea. The nucleotide sequences obtained were submitted to Database of the National Center for Biotechnology Information (NCBI) to nucleotide sequence access numbers assignment.

### In silico analysis of DNA sequences obtained

All the sequences obtained were analyzed using the *Bioedit 7.1* software and corroborated at GenBank using BlastN algorithm. The sequences were aligned against those available at database of the NCBI. The analysis was based on the similarity with the sequences aligned in the BLAST.

### Statistical analysis

The statistical analyses were performed using STATGRAPHICS Centurion XV.II. The chi-square test was used to examine association between serotypes distribution and years and virulence genes and serotypes. Evidence against the null hypothesis was considered with *p* values< 0.05.

## Results

### Colonization in full-term pregnant women

**Fig. 2:**
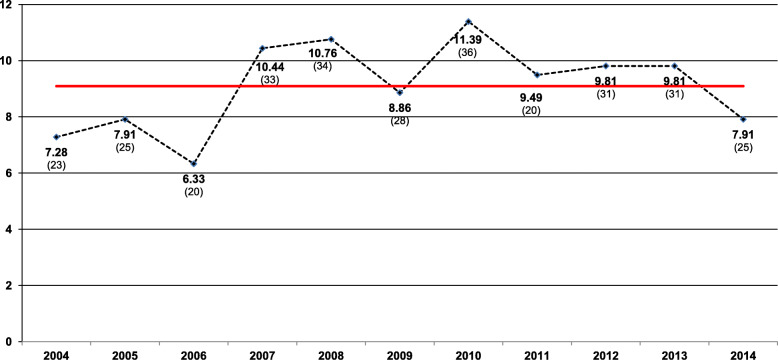
Prevalence of *S. agalactiae* colonization from women of 35–37 weeks of gestational age (*n* = 3480) per year. Misiones, Argentina, 2004–2014. The red line represents the overall prevalence. The numbers in parentheses represent the isolates obtained

Over the 11-years period, 3480 full-term pregnant women were studied and 316 GBS strains were recovered, an average of 310 samples were collected per year. The overall prevalence was 9.09%; with the lowest being 6.33% and the highest 11.39%, for the years 2006 and 2010, respectively (Fig. 2).

### Serotype determination

**Fig. 3:**
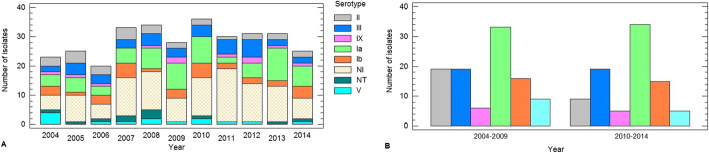
**a** Distribution of serotypes of *S. agalactiae* by year (2004–2014). **b** Distribution of serotypes in two time periods (2004–2009 and 2010–2014) (*n* = 189*). Misiones, Argentina. NI: not included in serotyping tests. NT: non-serotypeable * NT strains were excluded

We determined the serotypes of 200 colonizing GBS strains. The serotypes found were: Ia (67 isolates, 33.50%), III (38, 19.00%), Ib (31, 15.50%), II (28, 14%), V (14, 7.00%) and IX (11 strains, 5.50%). We did not detect serotypes IV, VI, VII and VIII. 5.50% of isolates were found to be non-serotypeable (NT) by the serological methods used. The most prevalent serotypes (Ia, Ib, II and III) were detected in all years. Meanwhile, the least prevalent (V and IX) were not detected in all years (Fig. 3a).

Due to the relatively small frequencies of some serotypes in some years, trends in serotypes by year could not be discerned. We evaluated the frequency of serotypes in two time periods: 2004–2009 and 2010–2014. We did not find statistically significant differences between these two periods (*p* = 0.5956). Therefore, we established that there are no changes in the distribution of serotypes over the years (Fig. 3b).

### Molecular detection of virulence-associated genes

**Fig. 4:**
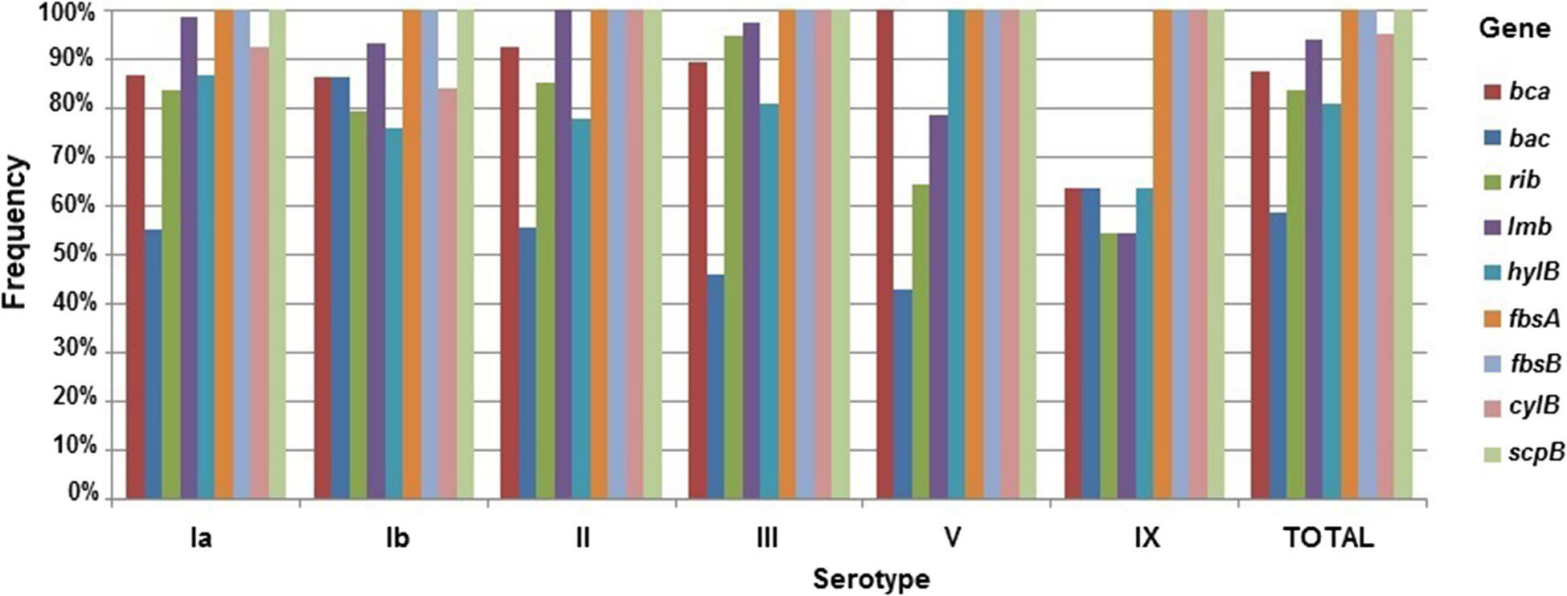
Frequency of virulence-associated genes and serotypes in *S. agalactiae* (*n* = 189*). Misiones, Argentina, 2004–2014. *NT strains were excluded

The *scp*B, *fbs*A and *fbs*B genes were detected in all the isolates, the other genes were detected in more than 80.00% of the isolates, except the *bac* gene (58.50%) (Table 2 and Fig. 4).

**Table 2:** Frequency of virulence-associated genes and serotypes in *S. agalactiae*. Misiones, Argentina, 2004–2014

Serotype	Virulence genes									n
	*bca*	*bac*	*rib*	*lmb*	*hylB*	*fbsA*	*fbsB*	*cylB*	*scpB*	
Ia	86.6% 58	55.2% 37	83.6% 56	98.5% 66	86.6% 58	100% 67	100% 67	92.5% 62	100% 67	67
Ib	86.2% 27	86.2% 27	79.3% 25	93.1% 29	75.9% 24	100% 31	100% 31	83.9% 26	100% 31	31
II	92.6% 26	55.6% 16	85.2% 24	100% 28	77.8% 22	100% 28	100% 28	100% 28	100% 28	28
III	89.2% 34	45.9% 18	94.6% 36	97.3% 37	81.1% 31	100% 38	100% 38	100% 38	100% 38	38
V	100% 14	42.8% 6	64.3% 9	78.6% 11	100% 14	100% 14	100% 14	100% 14	100% 14	14
IX	63.6% 7	63.6% 7	54.5% 6	54.5% 6	63.6% 7	100% 11	100% 11	100% 11	100% 11	11
TOTAL	87.8% 166	58.7% 111	82.5% 156	93.6% 177	83.6% 158	100% 189	100% 189	94.7% 179	100% 189	189

n (number of isolated investigated)

**Fig. 5:**
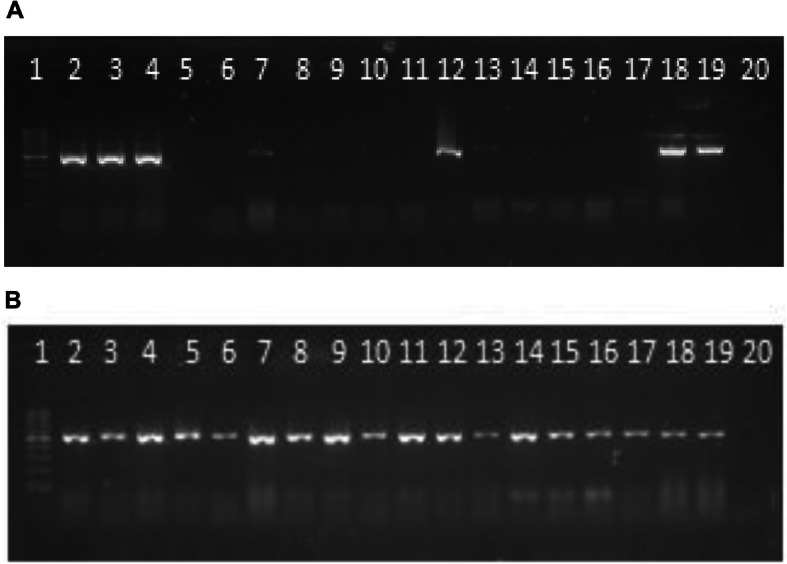
Electrophoresis in agarose gel 2% of PCR products for *bac* (**a**) and *bca* (**b**) genes in *S. agalactiae*. First lane, molecular weight marker, DNA Molecular Weight (D0017) 100 to 1000 bp-INBIO, Argentina —lane 2 to 18 strains serotype Ia: 23, 48, 96, 99, 74, 155, 165, 211, 213, 297, 390, 349, 489, 500, 835, 833, 1057; lane 19, positive control; last lane, negative control

The most common profiles were the ones including all genes (73 occurrences, 36.50%) and the one containing all genes except for *bac* (43 occurrences, 21.50%) (Fig. 5).

Since *scpB*, *fbsA*, and *fbsB* genes were detected in all isolates, they are homogeneously distributed among all serotypes. The statistical test of independence determined that there are no associations between the distribution of genes *bca* (*p* = 0.12) and *hylB* (*p* = 0.20) and the different serotypes. There were associations between serotype and genes *bac* (*p* = 0.003), *cylB* (*p* = 0.02), *rib* (*p* = 0.01) and *lmb* (*p* < 0.001).

### In silico analysis of DNA sequences obtained

Table 3 shows the size of the DNA fragments obtained for each gene investigated, the identity percentage using BLAST search and the Genbank access number of the sequence against which most similarity was obtained.

**Table 3:** In silico analysis of nucleotide sequences obtained for virulence-associated genes in *S. agalactiae* strains

Genes	Amplicon (bp)	Identity of sequences (%)	GenBank
*bac*	530	100%	CP0038110.1
*bca*	519	100%	CP007631.2
*rib*	369	100%	U58333.1
*hylB*	240	100%	NC004116.1
*lmb*	300	100%	NC004116.1
*cspB*	198	100%	U56908.1
*cylB*	148	99%	CP006910.1
*fbsA*	128	97%	CP006910.1
*fbsB*	160	99%	CP007632.1

*bp* base pairs

### Nucleotide sequence accession numbers

The nucleotide sequences reported in this paper have been submitted to the GenBank/EMBL sequence database and assigned accession numbers MN718728 (for the *bac* gene from *S. agalactiae* strain 264), MN725039, MN725043 and MT188756 (*bca, lmb* and *hylB* genes from *S. agalactiae* 221), MN725040, MN725041 MN725042, MN725044 and MT185681 (for *cylB, fbsA*, *fbsB*, *rib* and *scpB* genes from *S. agalactiae* 43BB).

## Discussions

### Colonization in full-term pregnant women

The frequency of vaginal-rectal colonization varies widely among geographic areas and even in different regions of the same country. Authors such as Ramos et al. (2009) suggest that; besides ethnic, geographic and social differences in the populations studied; the percentages of maternal colonization can be influenced by the different methods used to detect the bacteria [20].

The province of Misiones has the particularity that ethnic origins are varied, mainly from European immigrants and from neighboring countries. Since it shares more than 80% of its borders with Paraguay and Brazil, variable colonization figures are expected with respect to other Argentine provinces.

Our result of maternal colonization was 9.09%. The lowest prevalence was detected in 2006 (6.33%) and the highest in 2010 (11.39%). These values are similar to that reported by Oviedo et al (2013) (9.38%) using swab samples taken from the same study population, and they are within the range reported in Argentina: 1.40–18.20% [23, 31, 32]. Toresani et al. (2001) reported 3.20% for the city of Rosario [33]; Bavdaz et al. (2003) informed 5.20% for the city of Bariloche [34]; Cotainich et al. (2003) informed between 7.52 and 18.20% for the city of Cordoba [32], and García et al. (2003) between 5.40 and 17.80% for the province of Buenos Aires [35].

The average prevalence reported in South America is 15.90% [36]. Several studies in bordering countries reported similar colonization rates: in Brazil values from 4.20 to 28.40% [37]; in Chile, 19.80% [38]; and in Uruguay, 17.30% [39]. In Mexico, a literature review study found a 9.50% maternal colonization [40].

This research made it possible to determine the prevalence of maternal colonization, contributing to epidemiological surveillance in public health and adding data of interest at the national and regional levels. In addition, given the low to moderate prevalence values that we found, we recommend conducting the search in all pregnant women for the correct implementation of intrapartum antibiotic prophylaxis (IPP).

### Serotype determination

We detected serotypes Ia (33.50%), III (19.00%), Ib (15.50%), II (14.00%), V (7.00%), IX (5.50%) and 5.50% turned out to be non-serotypeable (NT). The most prevalent serotypes were detected in all years. Meanwhile, the least prevalent serotypes were not detected in all years (V in 2005 and 2013 and IX in 2007 and 2010). Serotypes IV, VI, VII and VIII were not detect in this study, results that agree those obtained Oviedo et al. (2013) who detected the same serotypes in 88 maternal isolates.

The percentage of NT strains found resembles data reported by Liébana-Martos et al. (2015), who detected 6.40% of these isolates [41]. This lack of reactivity with the agglutination test may be due to poor expression of the capsule.

Afshar et al. (2011) identify cross-reactions with the determination of serotypes V and IX with the reagents (Statens Serum Institute, Copenhagen, Denmark) used for the agglutination tests [42]. These investigations indicate that the accuracy of the results is highly dependent on experience, and the agglutination test with latex particles turns out to be less sensitive. Likewise, PCR-based capsule gene characterization methods accurately identify strains. However, studies conducted by Poyart et al. (2007) and Imperi et al. (2010) reported molecular methods that erroneously identified some strains [43, 44]. Therefore, the use of two different techniques was suggested for special cases of NT strains [41].

In the present research, the serotype Ia and III prevalences are like those conducted in Argentina on invasive isolations by Lopardo et al. in 2003 and by Perez et al. in 2004, on 58 and 66 isolates, respectively. Lopardo et al. (2003) detected serotype Ia (25.86%) and serotype III (22.41%) [45]. As Perez et al. (2004) recorded prevalences of serotype Ia (32.00%) and III (20.00%) [46].

Our results resemble also studies conducted in Chile by Martínez et al. (2004) and by Rojo et al. (2008) on 100 and 58 isolates, respectively. They detected: serotype III 36.90 and 56.90%, Ia 30.70 and 31.00%, II 21.5 and 8.60%, Ib 4.60 and 1.72% and of serotype V 1.50 and 1.70% [47, 48].

Our data differ from the results found in a multicenter study conducted in Brazil and published in 2014, in which, although the highest frequency corresponded to serotype Ia (27.60%), 8.10% of the isolates belonged to serotype IV [49].

Our results highlight that there have not been temporal changes in GBS serotype distribution in the province of Misiones. Nevertheless, since the dominant serotypes causing disease varies regionally, and even temporally, and differs by invasive and colonizing isolates [8, 27], the epidemiological monitoring of serotypes is important in a region because it contributes to vaccine design and the implementation of vaccination strategies in the future. As well as, to identify potential outbreaks. However, the application of serotyping alone is insufficient in epidemiologic scenarios in which molecular subtyping methods are necessary [50].

### Molecular detection of virulence-associated genes

The *scp*B, *fbs*A and *fbs*B genes were detected in all the isolates. The other genes were detected in more than 80.00% of the isolates, except the *bac* gene (58.50%).

The most common genotypic profiles were the ones including all the nine genes (73 occurrences, 36.50%) and the one containing all genes except for *bac* (43 occurrences, 21.50%). Oviedo et al. (2013), found that 28.40% of the strains simultaneously possessed the five genes investigated (*bca*, *bac*, *rib, lmb* and *hylB)*. The *lmb* (94.30%), *bca* (88.60%), *hylB* (79.50%) and *rib* (76.10%) genes were present in most of the isolates. The *bac* gene was found in 52.30% of the isolates. These results are similar, in spite of that they included a less amount of genes and isolates in their work [23].

We found associations between serotypes and genes *bac*, *cylB*, *rib* and *lmb*, which means that these genes are not distributed uniformly across the serotypes. However, we did not find an association between the other serotypes and virulence genes. Similar findings were detected by authors such as Dore et al. (2003) and Manning et al., (2006) who found association statistically significant in *bac*, *bca*, *hyl*B and *rib* genes with different serotypes [51, 52]. Authors such as Martins et al. (2011) found associations between serotype Ib and *bca*, serotype II and *bca* and serotype III and *rib* [53].

Persson et al. (2008) and Smith et al. (2007) established that the virulence genes investigated had a homogeneous distribution among serotypes and no gene is limited to a single serotype [54, 55].

Regarding the *bac*, *bca* and *rib* genes, our results resemble those found in vaginal secretions by Souza et al. (2013), in which *bca* was the most frequent, a finding that may represent the potential risk of invasive disease, given that the Cα protein contributes to the invasion of the cervical epithelium [10].

Our study differs from the figures observed by researchers such as Souza et al. (2013) and Dutra et al. (2014), who reported in Brazil percentages of *bac* and *bca* genes of 6.60 and 13.20% and 13.10 and 54.60% [10, 49].

The *scp*B gene coding for C5a peptidase was detected in 100% of the isolates studied. These results resemble those reported by Dutra et al. (2014) who have detected equal percentages of carrying the *scp*B gene. Other authors reported different frequencies for the *scp*B gene: 88.30% [56], 94.70% [57], 96.10% [58], and 97.60% [59], all equally high.

In our study, the genes *fbs*A and *fbs*B that encode both proteins have been detected in 100.00% of the strains studied. These results are like those reported by Rosenau et al. (2007) [60].

The *lmb* and *hyl*B genes were found in 94.00 and 81.00% of the isolates, respectively. This coincides with the assertion that they are found in most human isolates [61].

The importance of detecting virulence genes with respect to predicting the invasiveness of strains is contradictory. Smith et al. (2007) and Eskandarian et al. (2014) could not demonstrate any correlation between the virulence genes and clinical status of the patients from whom the isolates were obtained [54, 58]. In contrast, Manning et al. (2006) found that invasive strains were associated with specific serotype/gene combinations, but the association was only marginally significant. It is possible that the differences in pathogenicity are due to differences in gene expression [52].

We identified the nine genes investigated simultaneously in 73 of 200 colonizing isolates (36.50%). In a study carried out with strains that cause invasive diseases in the province of Misiones, all genes were detected in 72.73% of the isolates and it is observed that the strains recovered from neonates present all the virulence factors in percentages greater than 81.80%. However, the number of isolates analyzed was lower (*n* = 11) despite belonging to all invasive isolates recovered simultaneously during the years in which the present study was conducted [62].

Out of the nine virulence genes investigated in the colonizing GBS strains, *scp*B, *fbs*A and *fbs*B were detected more frequently. This suggests that the proteins they code could be included as antigenic epitopes in the development of a regional vaccine. However, the low frequency of *bac* gene (58.50%) detection would exclude it from this possibility.

The present findings, variables in the distribution of circulating serotypes with respect to other studies carried out in various countries; emphasize the need for permanent regional surveillance to contribute to the development of vaccines with adequate coverage for the region. As well as, to identify potential outbreaks and hypervirulent clones that require different observation in pregnancy or in the infant.

This research contributed to the prevention of the severe invasive disease caused by GBS during the years of the study, and no cases of neonatal infection were registered in the newborns of women in whom this microorganism was detected between 35 and 37 weeks of gestation, implementing intrapartum antibiotic prophylaxis according to the recommendations of national and international guidelines.

In addition, it allowed knowing the prevalence of maternal colonization and the distribution and circulating serotypes, contributing to epidemiological surveillance in public health and adding data of interest at the national and regional levels. This is the most comprehensive systematic study to date in Argentina, both for the number of GBS strains and for the number of virulence genes investigated.

## Data Availability

Sequences obtained in this study have been deposited in GenBank (Accession MN725039-MN725044, MN718728 and MT188756). Any other data referenced in this report is available from the corresponding author on reasonable request.

## Abbreviations

BE: Bile-Esculin test
BLAST: Basic local alignment search tool
CI: Confidence intervals
DNA: Deoxyribonucleic acid
EMBL: European Molecular Biology Laboratory
GBS: Group B *Streptococcus*
Hyp: Hippurate test
NCBI: National Center for Biotechnology Information
NT: Non serotypeable
PCR: Polymerase chain reaction
UBA: University of Buenos Aires

## References

[1] Jackson L, Hilsdon R, Farley M, Harrison L, Reingold A, Plikaytis B (1995). Risk factors for group B streptococcal disease in adults. Ann Intern Med.

[2] Centers for Disease Control and Prevention (CDC) (2007). Perinatal group B streptococcal disease after universal screening recommendations-United States, 2003–2005. MMWR Morb Mortal Wkly Rep.

[3] ARGENTINA, HONORABLE CONGRESO DE LA NACION. Ley Nacional 26.369. [on line] https://www.argentina.gob.ar/normativa/nacional/ley-26369-140274/texto.

[4] Misiones., Cámara de Representantes de la Provincia de. Digesto Jurídico. [on line] 2007. http://digestomisiones.gob.ar/buscador?page=92.

[5] Tudela C, Stewart R, Roberts S, Wendel G, Stafford I, McIntire D, Sheffield J (2012). Intrapartum evidence of early-onset group B *streptococcus*. Obstet Gynecol.

[6] Tamariz-Ortiz J, Obregón-Calero M, Jara-Aguirre J, Diaz-Herrera J, Jeferson-Cortez L, Guerra-Allison H (2004). Colonización vaginal y anorectal por *Streptococcus agalactiae* en gestantes de los Hospitales Nacionales Cayetano Heredia y Arzobispo Loayza. Rev Medica Hered.

[7] Seale A, Bianchi-Jassir F, Russell N (2017). Estimates of the Burden of Group B Streptococcal Disease Worldwide for Pregnant Women, Stillbirths and Children. Clin Infect Dis.

[8] Raabe V, Shane A, Fischetti V, Novick R, Ferretti J, Portnoy D, Braunstein M, Rood J (2019). Group B *Streptococcus* (*Streptococcus agalactiae*). Gram positive pathogens.

[9] Herbert M, Beveridge C, Saunders N (2004). Bacterial virulence factors in neonatal sepsis: group B *streptococcus*. Curr Opin Infect Dis.

[10] Souza V, Kegele F, Souza S, Neves F, de Paula G, Barros R (2013). Antimicrobial susceptibility and genetic diversity of *Streptococcus agalactiae* recovered from newborns and pregnant women in Brazil. Scand J Infect Dis.

[11] Larsson C, Lindroth M, Nordin P, Stålhammar-Carlemalm M, Lindahl G, Krantz I (2006). Association between low concentrations of antibodies to protein alpha and Rib and invasive neonatal group B streptococcal infection. Arch Dis Child Fetal Neonatal Ed.

[12] Ragunathan P, Sridaran D, Weigel A, Shabayek S, Spellerberg B, Ponnuraj K (2013). Metal binding is critical for the folding and function of laminin binding protein, Lmb of *Streptococcus agalactiae*. PLoS One.

[13] Devi A, Ponnuraj K (2010). Cloning, expression, purification and ligand binding studies of novel fibrinogen-binding protein FbsB of *Streptococcus agalactiae*. Protein Expr Purif.

[14] Pritzlaff C, Chang J, Kuo S, Tamura G, Rubens C, Nizet V (2001). Genetic basis for the beta-haemolytic/cytolytic activity of group B *Streptococcus*. Mol Microbiol.

[15] Wang Z, Guo C, Xu Y, Liu G, Lu C, Liu Y (2014). Two novel functions of hyaluronidase from *Streptococcus agalactiae* are enhanced intracellular survival and inhibition of proinflammatory cytokine expression. Infect Immun.

[16] Li Y, Zeng W, Li Y, Fan W, Ma H, Fan X (2019). Strecture determination of the CAMP factor of *Streptococcus agalactiae* with the aid of an MPB tag and insights into membrane-surface attachment. Acta Cryst.

[17] Hanson B, Runft D, Streeter C, Kumar A, Carion T, Neely M (2012). Functional analysis of the CpsA protein of *Streptococcus agalactiae*. J Bacteriol.

[18] Schubert A, Zakikhany K, Pietrocola G, Meinke A, Speziale P, Eikmanns B (2004). The fibrinogen receptor FbsA promotes adherence of *Streptococcus agalactiae* to human epithelial cells. Infect Immun.

[19] Lindahl G, Stålhammar-Carlemalm M, Areschoug T (2005). Surface proteins of *Streptococcus agalactiae* and related proteins in other bacterial pathogens. Clin Microbiol Rev.

[20] Ramos J, Milla A, López-García P, Gutiérrez F (2009). Estudio de colonización por *Streptococcus agalactiae* en gestantes extranjeras y españolas en Elche y Comarca. Enferm Infecc Microbiol Clin.

[21] Instituto Provincial de Estadísticas y Censos (IPEC). [on line] https://ipecmisiones.org/category/sociedad/indicadores-sociales/seguridad-publica/.

[22] Argentina.Ar. [on line] https://web.archive.org/web/20140712224945/http://www.argentina.ar/temas/turismo/19519-misiones-por-la-senda-de-los-inmigrantes.

[23] Oviedo P, Pegels E, Laczeski M, Quiroga M, Vergara M (2013). Phenotypic and genotypic characterization of *Streptococcus agalactiae* in pregnant women. First study in a province of Argentina. Braz J Microbiol.

[24] Rodriguez-Granger J, Alvargonzalez J, Berardi A, Berner R, Kunze M, Hufnagel M (2012). Prevention of group B streptococcal neonatal disease revisited. The DEVANI European project. Eur J Clin Microbiol Infect Dis.

[25] Madhi S, Dangor Z, Heath P, Schrag S, Izu A, Sobanjo-Ter Meulen A (2013). Considerations for a phase-III trial to evaluate a group B *Streptococcus* polysaccharide-protein conjugate vaccine in pregnant women for the prevention of early- and late-onset invasive disease in young-infants. Vaccine.

[26] Collin S, Lamb P, Jauneikaite E, Le Doare K, Creti R, Barardi A (2019). Hospital clusters of invasive group B streptococcal disease: a systematic review. J Inf Secur.

[27] Dangor Z, Cutland C, Izu A, Kwatra G, Trenor S, Lala S, Madhi S (2016). Temporal Changes in Invasive Group B Streptococcus Serotypes: Implications for Vaccine Development. PLoS ONE.

[28] Rozen S, Skaletsky HJ, Krawetz S, Misener S (2000). Primer3 on the WWW for general users and for biologist programmers. Bioinformatics Methods and Protocols: Methods in Molecular Biology.

[29] Sambrook J, Russell DW (2001). Molecular Cloning: a Laboratory Manual.

[30] Cariaga Martinez A, Zapata P (2007). Protocolos de Extracción de ADN. El Laboratorio de Biología Molecular. Ed Universitaria de Misiones.

[31] Larcher J, Capellino F, De Giusto R, Travella C, Balangione F, Kreiker G (2005). Group B *streptococcus* colonization during pregnancy and prevention of early onset of disease. Medicina (B Aires).

[32] Cotainich H (2003). Prevalencia de portación de estreptococo grupo B en gestantes provenientes de un servicio privado del interior del país. Reunión Científica Microbiología Clínica-SADEBAC. O-008.

[33] Toresani I, Limansky A, Bogado I, Guardati M, Viale A (2001). Phenotypic and genotypic study of *Streptococcus agalactiae* in vagina of pregnant women in Argentina. Medicina..

[34] Bavdaz B (2003). Screening prenatal de Estreptococo grupo B en Bariloche. Reunión Científica Mirobiología Clínica-*SADEBAC*. O-007.

[35] García S, Eliseth M, Lazzo M, Copolillo E, Barata A, de Torres R (2003). Group B *Streptococcus* carriers among pregnant women. Rev Argent Microbiol.

[36] Russell N, Seale A, O’Driscoll M, O’Sullivan C, Bianchi-Jassir F, Gonzalez-Guarin J (2017). Maternal Colonization With Group B Streptococcus and Serotype Distribution Worldwide: Systematic Review and Meta-analyses. Clin Infect Dis.

[37] Do Nascimento C, Dos Santos N, Ferreira R, Taddei C (2019). *Streptococcus agalactiae* in pregnant women in Brazil: prevalence, serotypes, and antibiotic resistance. Braz J Microbiol.

[38] Abarzua F, Guzman A, Belmar C, Becker J, García P, Rioseco A. Prevalencia de colonización por *Streptococcus agalactiae* (grupo B) en el tercer trimestre del embarazo. Evaluación del cultivo selectivo. Experiencia en 2192 pacientes. Rev Chil Obstet Ginecol. 2002;67(2). 10.4067/S0717-75262002000200001.

[39] Laufer J, Scasso S, Sosa C, Rodríguez-Cuns G, Alonso J, Pons J (2009). Group B *streptococcus* colonization among pregnant women in Uruguay. Int J Gynaecol Obstet.

[40] Reyna Figueroa J, Ortiz Ibarra F, Esteves Jaramillo A, Casanova RG (2007). Maternal group B *Streptococcus* colonization in Mexico: prevalence based on literature review. Ginecol Obstet Mex.

[41] Liébana-Martos M, Cabrera-Alavargonzalez J, Rodríguez-Granger J, Miranda-Casas C, Sampedro-Martínez A, Gutiérrez-Fernández J (2015). Serotypes and antibiotic resistance patterns in beta-hemolytic *Streptococcus* agalactiae isolates in colonized mothers and newborns with invasive disease. Enferm Infecc Microbiol Clin.

[42] Afshar B, Brougthon K, Creti R, Decheva A, Hufnagel M, Kriz P (2011). International external quality assurance for laboratory identification and typing of *Streptococcus agalactiae* (group B streptococci). J Clin Microbiol.

[43] Poyart C, Tazi A, Réglier-Poupet H, Billoët A, Tavares N, Raymond J, Trieu-Cuot P (2007). Multiplex PCR assay for rapid and accurate capsular typing of group B streptococci. J Clin Microbiol.

[44] Imperi M, Pataracchia M, Alfarone G, Baldassarri L, Orefici G, Creti R (2010). A multiplex PCR assay for the direct identification of the capsular type (Ia to IX) of *Streptococcus agalactiae*. J Microbiol Methods.

[45] Lopardo H, Vidal P, Jeric P, Centron D, Paganini H, Facklam R (2003). Six-month multicenter study on invasive infections due to group B streptococci in Argentina. J Clin Microbiol.

[46] Pérez J, Limansky A, Toresani I, Ebner G, Di Bartolomeo S, de Inocente I (2004). Distribución de tipo capsular y sensibilidad a antimicrobiana de *Streptococcus agalactiae* productores de infecciones en Argentina. Rev Argent Microbiol.

[47] Martínez M, Ovalle A, Durán C, Reid I, Urriola G, Garay B (2004). Serotypes and antimicrobial susceptibility of *Streptococcus agalactiae*. Rev Med Chil.

[48] Rojo P, Araya P, Martínez T, Hormazábal J, Maldonado A, Fernández J (2008). Molecular characterization of Chilean isolates of *Streptococcus agalactiae*. Rev Med Chil.

[49] Dutra V, Alves V, Olendzki A, Dias C, de Bastos AF, Santos G (2014). *Streptococcus agalactiae* in Brazil: serotype distribution, virulence determinants and antimicrobial susceptibility. BMC Infect Dis.

[50] MacFarquhar J, Jones T, Woron A, Kainer M, Whitney C, Beall B (2010). Outbreak of late-onset group B *Streptococcus* in a neonatal intensive care unit. Am J Infect Control.

[51] Dore N, Bennett D, Kaliszer M, Cafferkey M, Smyth C (2003). Molecular epidemiology of group B streptococci in Ireland: associations between serotype, invasive status and presence of genes encoding putative virulence factors. Epidemiol Infect.

[52] Manning S, Ki M, Marrs C, Kugeler K, Borchardt S, Baker C, et al. The frequency of genes encoding three putative group B streptococcal virulence factors among invasive and colonizing isolates. BMC Infect Dis. 2006;6(116). 10.1186/1471-2334-6-116. 10.1186/1471-2334-6-116 PMC1559624 16846499

[53] Martins E, Andreu A, Correia P, Juncosa T, Bosch J, Ramirez M (2011). Group B streptococci causing neonatal infections in Barcelona are a stable clonal population: 18-year surveillance. J Clin Microbiol.

[54] Smith T, Roehl S, Pillai P, Li S, Marrs C, Foxman B (2007). Distribution of novel and previously investigated virulence genes in colonizing and invasive isolates of *Streptococcus agalactiae*. Epidemiol Infect.

[55] Persson E, Berg S, Bevanger L, Bergh K, Valsö-Lyng R, Trollfors B (2008). Characterisation of invasive group B streptococci based on investigation of surface proteins and genes encoding surface proteins. Clin Microbiol Infect.

[56] Udo E, Boswihi S, Al-Sweih N (2013). Genotypes and virulence genes in group B *streptococcus* isolated in the maternity hospital, Kuwait. Med Princ Pract.

[57] Hannoun A, Shehab M, Khairallah M, Sabra A, Abi-Rached R, Bazi T (2009). Correlation between group B streptococcal genotypes, their antimicrobial resistance profiles, and virulence genes among pregnant women in Lebanon. Int J Microbiol.

[58] Eskandarian N, Ismail Z, Neela V, van Belkum A, Desa M, Amin NS (2014). Antimicrobial susceptibility profiles, serotype distribution and virulence determinants among invasive, non-invasive and colonizing *Streptococcus agalactiae* (group B *streptococcus*) from Malaysian patients. Eur J Clin Microbiol Infect Dis.

[59] Beigverdi R, Jabalameli F, Mirsalehian A, Hantoushzadeh S, Boroumandi S, Taherikalani M, Emaneini M (2014). Virulence factors, antimicrobial susceptibility and molecular characterization of *Streptococcus agalactiae* isolated from pregnant women. Acta Microbiol Immunol Hung.

[60] Rosenau A, Martins K, Amor S, Gannier F, Lanotte P, van der Mee-Marquet N (2007). Evaluation of the ability of *Streptococcus agalactiae* strains isolated from genital and neonatal specimens to bind to human fibrinogen and correlation with characteristics of the *fbsA* and *fbsB* genes. Infect Immun.

[61] Al Safadi R, Amor S, Hery-Arnaud G, Spellerberg B, Lanotte P, Mereghetti L (2010). Enhanced expression of *lmb* gene encoding laminin-binding protein in *Streptococcus agalactiae* strains harboring IS1548 in *scpB-lmb* intergenic region. PLoS One.

[62] Laczeski M, Novosak N, Cannistraci Giolito R, Littvik A, Paván J, Villalba V, Vergara M (2015). Study of serotypes, susceptibility to macrolide and virulence and resistance molecular profiles in invasive strains of *Streptococcus agalactiae* in two Argentine provinces. Adv Microbiol.

